# Sheng Mai San Mitigates Heat Stress-Induced Myocardial Injury by Coordinated Regulation of the Keap1-Nrf2-HO-1 and Stub1-HSF1 Signaling Pathways

**DOI:** 10.3390/antiox14091140

**Published:** 2025-09-22

**Authors:** Jiaqi Dong, Qian Ma, Rong Yang, Xiaosong Zhang, Yongli Hua, Peng Ji, Wanling Yao, Ziwen Yuan, Yanming Wei

**Affiliations:** 1 College of Veterinary Medicine, Gansu Agricultural University, Lanzhou 730070, China; 18893811442@163.com (J.D.);; 2 College of Veterinary Medicine, China Agricultural University, Beijing 100091, China

**Keywords:** Sheng Mai San, heat stress, cardiac injury, Keap1-Nrf2-HO-1, Stub1-HSF1

## Abstract

Heat stress (HS), a pervasive environmental stressor, significantly disrupts systemic physiological homeostasis, posing substantial threats to human and animal health. Sheng Mai San (SMS), a classic Traditional Chinese Medicine (TCM) formula, exerts its therapeutic effects by replenishing qi (the vital energy governing physiological functions) and nourishing yin (the material basis responsible for moistening and cooling actions). This formula demonstrates significant efficacy in astringing sweating and preventing collapse. However, its precise molecular mechanisms against HS-induced myocardial injury remain incompletely elucidated. This study initially employed physicochemical analytical methods to determine the contents of total polysaccharides, saponins, and flavonoids in SMS and evaluated its antioxidant activity. Subsequently, both in vitro and in vivo rat models of HS were established to systematically assess the alterations in reactive oxygen species (ROS), antioxidant enzymes (GSH, SOD, CAT), and heat shock proteins (HSP70, HSP90) following SMS intervention, thereby investigating HS-induced myocardial injury and the protective effects of SMS. Furthermore, Western blot, immunofluorescence, and qRT-PCR techniques were utilized to quantitatively analyze key molecules in the Keap1-Nrf2-HO-1 and Stub1-HSF1 signaling pathways. The results demonstrated that total polysaccharides were the most concentrated in SMS, followed by total saponins. This formula exhibited potent free radical scavenging capacity against DPPH, ABTS, and OH^−^, along with significant reducing activity. HS-induced myocardial injury reached its peak severity at 6-12 h post-stress exposure. SMS intervention effectively suppressed excessive ROS generation, enhanced the activities of antioxidant enzymes (GSH, SOD, and CAT), and downregulated HSP70 and HSP90 mRNA expression levels, thereby significantly mitigating cardiomyocyte damage. Mechanistic investigations revealed that SMS conferred cardioprotection through dual modulation of the Keap1-Nrf2-HO-1 and Stub1-HSF1 signaling pathways. This study not only provides a novel TCM-based therapeutic strategy for preventing and treating HS-related cardiovascular disorders but also establishes a crucial theoretical foundation for further exploration of SMS’s pharmacological mechanisms and clinical applications.

## 1. Introduction

With the ongoing progression of global warming, the frequency and intensity of extreme heat events have shown a significant upward trend, posing unprecedented challenges to human health [1]. HS refers to the physiological response that occurs when organisms are chronically exposed to high-temperature environments (often accompanied by high humidity), resulting in cumulative body heat exceeding thermolytic capacity [2]. This condition emerges when impaired thermoregulatory function causes abnormal elevation of core body temperature. The key mechanism lies in its detrimental effects on evaporative cooling through perspiration and respiration, where high humidity reduces the water vapor pressure gradient between skin and environment, thereby impeding heat dissipation through sweat evaporation [3]. Epidemiological studies have confirmed a distinct J/U-shaped association between heat exposure and mortality [4]. It should be specifically noted that the disease burden and mortality data cited in this study refer specifically to health impairments directly caused by heat exposure, with evidence primarily derived from large-scale epidemiological investigations of extreme heat events and high-temperature occupational populations [5,6,7,8]. This study focuses on pathological states directly induced by environmental and occupational heat exposure, with particular emphasis on analyzing the pathogenesis of multi-organ dysfunction and related diseases resulting from heat stress. Research indicates that susceptible populations (such as the elderly and patients with cardiovascular diseases) are more prone to developing severe heat-related illnesses after heat exposure; meanwhile, high-temperature occupational groups including athletes and construction workers, due to long-term high-intensity work, exhibit significantly higher incidence risks and clinical severity of heat-related diseases compared to the general population [1]. These findings not only reveal the significant public health threats posed by climate warming but also highlight the strategic importance of advancing research on HS mechanisms. The development of targeted prevention and intervention strategies is of critical practical significance for protecting vulnerable populations and enhancing societal resilience to climate change.

Extensive studies have demonstrated that suppressing oxidative stress represents a pivotal therapeutic strategy for preventing and treating HS-related disorders. Under hyperthermic conditions, myocardial tissues exhibit abnormal accumulation of ROS. These excessive ROS molecules attack critical biomacromolecules including nucleic acids and proteins within cardiomyocytes, inhibit the activity of key lipid-metabolizing enzymes, and significantly reduce the biological activity of antioxidant enzymes such as superoxide dismutase (SOD), ultimately leading to myocardial oxidative damage and even cellular apoptosis [9]. Nuclear factor erythroid 2-related factor 2 (Nrf2), a cap-n-collar basic region-leucine zipper (bZIP) transcription factor, serves as the master regulator of cellular antioxidant defense systems [10]. Under physiological conditions, Nrf2 is maintained in a low-activity state through its binding to Kelch-like ECH-associated protein 1 (Keap1) in the cytoplasm. Keap1 serves as the substrate recognition subunit of the Cullin3-RBX1 E3 ubiquitin ligase complex, exhibiting dual functionality as both a sensor of oxidative/electrophilic stress and a regulator that maintains low basal Nrf2 expression levels through ubiquitin-mediated proteasomal degradation [11,12]. Under oxidative stress conditions, ROS induce conformational changes in Keap1 through specific modifications of its critical cysteine residues, thereby destabilizing the Keap1-Cullin3-RBX1 complex. This process facilitates nuclear translocation of Nrf2, which subsequently forms heterodimers with small MAF (sMAF) proteins in the nucleus. The Nrf2-sMAF complex then binds to antioxidant response elements (AREs), initiating transcription of various cytoprotective genes [13,14]. In a broiler HS model, Wu Yanan’s research team demonstrated that HS impairs the Keap1-Nrf2-ARE signaling pathway, as evidenced by downregulation of downstream target genes (GCLC and NQO1), decreased serum activities of SOD and glutathione, and elevated levels of malondialdehyde (MDA), a lipid peroxidation product [9]. These findings provide crucial experimental evidence for elucidating the molecular mechanisms underlying HS-induced myocardial injury.

During the cellular response to HS, the cardiac tissue-specific molecular chaperone Stub1 (carboxyl terminus of Hsc70-interacting protein, CHIP) plays a pivotal regulatory role [15]. Under physiological conditions, Stub1 is predominantly localized in the cytoplasm, with detectable but minimal nuclear presence. Upon HS exposure, Stub1 undergoes rapid nuclear translocation and significantly enhances its binding affinity for heat shock proteins (HSPs), including HSP70 and HSP90 [16]. However, under conditions of excessive stress, nuclear Stub1 levels exhibit paradoxical downregulation [17]. This biphasic regulation is mediated through the heat shock transcription factor 1 (HSF1), which serves as the master molecular switch connecting environmental stress with HSP induction [18]. Mechanistically, activated HSF1 forms functional trimers that bind to heat shock elements (HSEs) in the promoters of HSP genes, thereby initiating their transcriptional activation [19]. Emerging evidence demonstrates that Stub1 not only regulates the chaperone system but also directly facilitates HSF1 activation and nuclear translocation [20]. Mechanistically, Stub1 promotes HSP70-HSF1 complex dissociation, enhances HSF1 trimerization, and co-localizes with HSF1 in the nucleus to potentiate its DNA-binding activity, thereby upregulating HSP70 expression to counteract misfolded protein accumulation [20]. Therefore, elucidating the regulatory mechanisms of the Stub1-HSF1 signaling pathway under HS conditions and its cardioprotective effects holds significant theoretical importance and provides novel therapeutic perspectives for clinical prevention and treatment of HS-induced myocardial injury.

SMS, a classical Chinese herbal formula originally documented in “Yixue Qiyuan” by Zhang Yuansu during the Jin Dynasty, is clinically prescribed for replenishing qi, nourishing yin, and arresting sweating [21,22]. Contemporary pharmacological studies have demonstrated its significant therapeutic effects in improving cardiac function, alleviating myocardial ischemia, enhancing hypoxia tolerance, and protecting cardiomyocytes [23,24]. Our previous phytochemical analysis identified ginsenosides Rb1, Rh1, and Rg3; ophiopogonin D; ruscogenin; and schisandrin A as the major bioactive constituents of SMS, with ginsenoside Rg3 and schisandrin A being the most abundant components [25]. Ginsenoside Rg3 activated the Keap1/Nrf2 signaling pathway to attenuate OGD/R-induced ferroptosis in H9c2 cells [26]. Similarly, schisandrin A has been shown to suppress RANKL-induced ROS generation via Nrf2 activation and concurrently inhibit NF-κB signaling, thereby effectively attenuating osteoclast differentiation [27]. To elucidate the cardioprotective mechanisms of SMS against HS-induced myocardial injury, we quantitatively analyzed its contents of total polysaccharides, total flavonoids, and total saponins; systematically evaluated its in vitro antioxidant capacity; and established both in vitro and in vivo HS models. By employing multiple techniques including immunofluorescence, Western blotting, and quantitative real-time polymerase chain reaction (qRT-PCR), we comprehensively investigated the regulatory effects of SMS on the Keap1-Nrf2-HO-1 and Stub1-HSF1 signaling pathways, providing novel therapeutic strategies for the clinical prevention and treatment of HS-induced myocardial injury.

## 2. Materials and Methods

### 2.1. Reagents

Catalase (CAT) (Cat. No.: A007-2-1), SOD (Cat. No.: A001-3-2), and glutathione (GSH) (Cat. No.: A006-2-1) were purchased from Nanjing Jiancheng Bioengineering Institute (Nanjing, China). An ROS assay kit (Cat. No. S0033S) and N-acetyl-L-cysteine (NAC) (Cat. No. ST1546-50g) were obtained from Beyotime Biotechnology (Shanghai, China). The Stub1 antibody (Cat. No.: ab134064) was acquired from Abcam (Cambridge, UK). HSP70 (Cat. No.: WL01019) and HSF1 (Cat. No.: WL20759) antibodies were procured from Wanleibio Co., Ltd. (Shenyang, China). Nrf2 (Cat. No.: 16396-1-AP), Keap1 (Cat. No.: 10503-2-AP), GAPDH (Cat. No.: 10494-1-AP), Laminb1 (Cat. No.: 12987-1-AP), and HO-1 (Cat. No.: 10701-1-AP) antibodies were purchased from Proteintech Group (Wuhan, China).

### 2.2. Preparation of SMS Extract

The herbal components of SMS, including *Panax ginseng* C.A. Mey., *Ophiopogon japonicus* (L.f.) Ker-Gawl., and *Schisandra chinensis *(Turcz.) Baill., were weighed at a ratio of 3:3:2, respectively. The mixture was ground using a pulverizer and sieved through a 20-mesh screen. Subsequently, the powder was soaked in 10 volumes of distilled water for 30 min, followed by decoction—initially brought to a boil over high heat, then simmered at low heat for 60 min. The resulting filtrate was collected, and the residue was subjected to a second extraction with 8 volumes of distilled water for 40 min. After filtration, the two extracts were combined and centrifuged at 3000 r/min for 15 min. The supernatant was concentrated under reduced pressure at 60 °C to achieve a final concentration of 1.0 g crude herb/mL. The concentrated solution was then freeze-dried to obtain the SMS freeze-dried powder [28,29].

### 2.3. Chemical Composition Analysis of SMS

A total of 500 mg of accurately weighed lyophilized SMS powder was dissolved in 50% ethanol to a final volume of 5 mL, followed by ultrasonication for 30 min and centrifugation. The supernatant was filtered through a 0.22 μm membrane prior to analysis. Standard solutions of glucose, rutin, and ginsenoside Rg3 were prepared at serial concentrations for quantitative determination. The contents of total polysaccharides, total flavonoids, and total saponins were measured using modified colorimetric methods: phenol-sulfuric acid assay [30], sodium nitrite-aluminum nitrate-sodium hydroxide assay [31], and vanillin-perchloric acid assay [32], respectively. Methodological validation was performed to verify the reliability of the analytical procedures.

### 2.4. In Vitro Antioxidant Activity Assay

The antioxidant capacity of SMS was systematically evaluated using four standard assays: DPPH radical scavenging (517 nm) [33], ABTS radical scavenging (734 nm) [33], OH^−^ radical scavenging (520 nm) [34], and ferric ion reducing power assessment [potassium ferricyanide method (700 nm) and FRAP assay (593 nm)] with NAC as the positive control. Measurements were performed using UV-visible spectrophotometry, with each experiment independently repeated in triplicate. Radical scavenging rates were calculated according to standard formulas.

### 2.5. Cell Experiments

#### 2.5.1. Cell Culture

Rat H9c2 cardiomyocytes were purchased from Wuhan Procell Life Science & Technology Co., Ltd. (Wuhan, China). The cells were cultured in DMEM complete medium, consisting of 79% DMEM, 20% fetal bovine serum (FBS), and 1% penicillin-streptomycin-amphotericin B, and maintained in a humidified incubator at 37 °C with 5% CO_2_. Subculturing was performed when cells reached 70% confluence.

#### 2.5.2. Establishment of H9c2 Cardiomyocytes HS Model

H9c2 cardiomyocytes were seeded in culture plates and divided into the following groups: Control, HS-0 h, HS-3 h, HS-6 h, HS-9 h, and HS-12 h. The Control group was maintained under standard culture conditions (37 °C, 5% CO_2_) throughout the experiment. The remaining groups were subjected to HS (43 °C, 5% CO_2_) for 2 h, followed by recovery at 37 °C, 5% CO_2_ for 0, 3, 6, 9, or 12 h, respectively.

#### 2.5.3. SMS Intervention and Grouping

H9c2 cardiomyocytes were seeded in culture plates and allowed to adhere for 24 h prior to drug treatment. The experimental groups were treated with different concentrations of SMS (500, 1000, and 2000 µg/mL) or NAC (0.125 µM). Additionally, intervention groups were established using YL-109 (a Stub1 agonist, MCE) or Curcumin (an Nrf2 agonist, MCE). Following 24 h of drug treatment, all groups were subjected to HS (43 °C, 5% CO_2_) for 2 h, followed by recovery under standard conditions (37 °C, 5% CO_2_).

### 2.6. Animal Experiment

Sixty Sprague-Dawley (SD) rats were obtained from the Lanzhou Veterinary Research Institute of Chinese Academy of Agricultural Sciences [license number: SCXK (Gan) 2020-0002]. The rats were acclimatized for 7 days in standard housing cages under controlled conditions: 12 h light/dark cycle, ambient temperature of 23 ± 1 °C, and relative humidity of 45 ± 5%. Throughout the study, animals had ad libitum access to standard rodent chow and drinking water. The HS model was established according to a previously described protocol [35] with modifications to better approximate clinical conditions. Briefly, SD rats were placed in a preheated climate-controlled chamber and subjected to daily HS exposure (38 ± 1 °C, 75 ± 5% relative humidity) for 2 h per day (from 10:00 to 12:00) over 7 consecutive days, during which food and water were withheld. Control animals were maintained under standard conditions (23 ± 1 °C, 45 ± 5% relative humidity). The temperature and humidity parameters were selected based on the following rationales: pilot studies confirmed that this specific combination effectively induced significant hyperthermia (core body temperature elevated to 40.5–41.5 °C) without causing acute mortality, thereby closely mimicking the clinical manifestation of heat stroke in human patients; furthermore, these conditions simulate the typical heatwave climate prevalent in southern China and reflect occupational heat exposure environments [6,7]; ultimately, this model reliably recapitulates key pathophysiological features observed in human heat stroke, including HSP70 upregulation, systemic inflammatory response, and multi-organ dysfunction. Two hours prior to each HS exposure, animals received the following treatments via oral gavage: the Control and HS groups were administered normal saline; the SMS-treated groups received SMS at high (SMS-H, 5.04 g/kg), medium (SMS-M, 2.52 g/kg), or low (SMS-L, 1.26 g/kg) doses; and the positive control group received NAC (150 mg/kg) [36]. Biological samples were collected 6 h after the final HS exposure.

### 2.7. Determination of CAT, GSH, SOD, and ROS Levels in H9c2 Cardiomyocytes

Following treatment, cells were lysed using RIPA buffer and centrifuged at 3000 r/min for 15 min. The supernatant was collected for protein concentration determination using the BCA method. Subsequently, intracellular levels of ROS, CAT, SOD, and GSH were measured strictly according to the manufacturer’s protocols of the respective assay kits.

### 2.8. Measurement of Intracellular ROS Levels in H9c2 Cardiomyocytes Using Fluorescent Probe

H9c2 cardiomyocytes were seeded in 6-well plates at a density of 2 × 10^5^ cells/mL (4 × 10^5^ cells/well in 2 mL medium) and allowed to adhere for 24 h. After experimental treatments, cells were washed three times with PBS and incubated with 1 mL DCFH-DA working solution in a 37 °C, 5% CO_2_ incubator for 20 min protected from light. Following three additional PBS washes to remove unincorporated probe, 1 mL serum-free medium was added prior to fluorescence microscopy observation and image acquisition. Three independent replicates were performed for each experimental group.

### 2.9. Quantitative Real-Time PCR Analysis

Total RNA was extracted using TRIzol reagent and reverse-transcribed into cDNA. Quantitative PCR was performed on a LightCycler 96 system using ChamQ Universal SYBR qPCR Master Mix, with all primers synthesized by Sangon Biotech (Shanghai). The primer sequences were as follows: HSP70: Forward: 5′-TCGAACAGTTCAAGATCAAACG-3′, Reverse: 5′-CCGGAACTTCTTGTAGTAGTCA-3′; HSP90: Forward: 5′-GAAGTGAACAAGCTTGATGGAG-3′, Reverse: 5′- GTCCACGCTATTTTCATCAGAC-3′; Stub1: Forward: 5′-TGAATGAGCTCTT-3′, Reverse: 5′-GTCACAGGATCGAAATGGCC-3′; HSF1: Forward: 5′- CCATGAAGCACGAGAACGAG-3′, Reverse: 5′- ACTGCACCAGTGAGATCAGGA -3′; GAPDH: Forward: 5′-ACGGGAAACCCATCACCATC-3′, Reverse: 5′-CTCGTGGTTCACACCCATCA-3′.

### 2.10. Immunofluorescence Analysis

For immunofluorescence staining, H9c2 cardiomyocytes from different treatment groups were processed as follows: Cells were washed three times with PBS, fixed with 4% paraformaldehyde at 37 °C for 15 min, and subsequently washed three times with PBS (5 min each). Permeabilization was performed using 0.1% Triton X-100 at room temperature for 15 min, followed by three additional PBS washes (5 min each). Non-specific binding was blocked with 3% bovine serum albumin (BSA) at 37 °C for 30 min. Primary antibodies against Nrf2 (1:200 dilution), Stub1 (1:500), and HSF1 (1:200) were applied and incubated overnight at 4 °C. The following day, cells were rewarmed to room temperature, washed three times with PBS (5 min each), and incubated with appropriate fluorescent secondary antibodies at 37 °C for 30 min. After three final PBS washes (5 min each), nuclei were counterstained with DAPI at room temperature, and slides were mounted for microscopic observation.

### 2.11. Western Blot Analysis

Nuclear proteins were extracted from cardiac tissues using a commercial nuclear protein extraction kit according to the manufacturer’s protocol. Protein concentrations were determined by BCA assay, and equal amounts of protein were loaded for electrophoresis. Proteins were separated on SDS-PAGE gels (80 V for 40 min followed by 100 V for 80 min) and subsequently transferred to PVDF membranes at 200 mA for 90 min. Membranes were blocked with 5% skim milk for 2 h at room temperature, then incubated overnight at 4 °C with the following primary antibodies: HSP70 (1:1000), Keap1 (1:3000), Nrf2 (1:3000), HO-1 (1:3000), Stub1 (1:10,000), HSF1 (1:1000), and GAPDH (1:2000). After 10 washes with TBST, membranes were incubated with appropriate horseradish peroxidase (HRP)-conjugated secondary antibodies for 1 h at room temperature. Following another 10 TBST washes, protein bands were visualized using ECL substrate and quantified using ImageJ software (version 1.53t).

### 2.12. Statistical Analysis

All experimental data were analyzed using one-way analysis of variance (ANOVA) followed by Tukey’s post hoc test for multiple comparisons. Results are presented as mean ± standard error of the mean (SEM). Statistical analyses and graphical representations were performed using GraphPad Prism 9 software (GraphPad Software, San Diego, CA, USA). A probability value of *p* < 0.05 was considered statistically significant.

## 3. Results

### 3.1. Chemical Composition of SMS

Quantitative analysis revealed that SMS contained the highest content of total polysaccharides (57.41%), followed by total saponins (10.98%) and total flavonoids (0.42%). Method validation demonstrated reliable determination of all components, with precision (RSD ≤ 3.56%), stability (RSD ≤ 3.34%), repeatability (RSD ≤ 4.20%), mean spike recovery (93.73–106.06%; RSD ≤ 3.69%), and validation test results (RSD ≤ 3.37%), confirming the suitability of this method for quality control of SMS. Notably, total polysaccharides were identified as the predominant active constituents (Table 1).

### 3.2. In Vitro Antioxidant Activity of SMS

**Figure 1:**
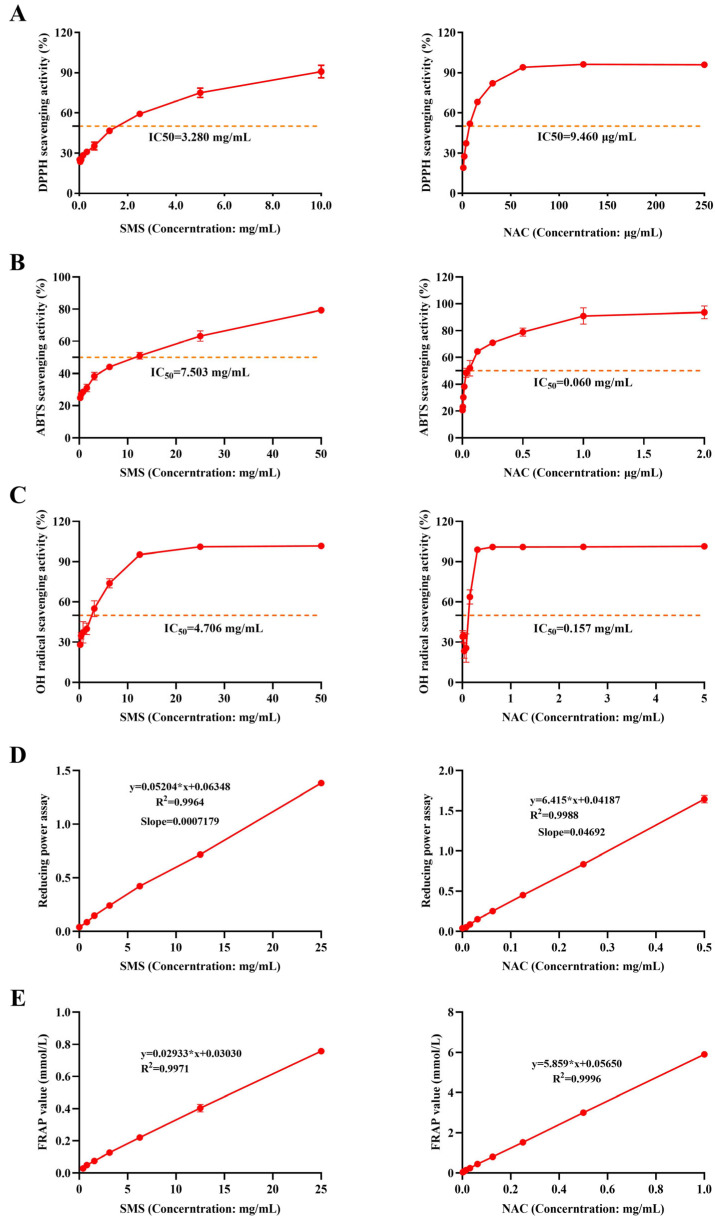
SMS exerts antioxidant effects by scavenging DPPH, ABTS, and hydroxyl radicals and enhancing reducing power. (**A**) DPPH scavenging rate; (**B**) ABTS scavenging rate; (**C**) OH-scavenging rate; (**D**) reducing power; (**E**) FRAP activity; “∗” represents multiplication in formulas (n = 3/group).

SMS exhibited a significant dose-dependent effect on DPPH free radical, ABTS free radical, and OH^−^ scavenging activities. The half maximal inhibitory concentration (IC50) of the SMS aqueous extract was 3.280 mg/mL for DPPH radicals, 7.503 mg/mL for ABTS radicals, and 4.706 mg/mL for hydroxyl radicals (Figure 1A–C). Additionally, the aqueous extract of SMS demonstrated a clear dose–response relationship for Fe^3+^ reducing power, with a slope of 0.0007179. At the same time, the FRAP method was used to evaluate the Fe^3+^ reduction ability. The Fe^3+^ reduction capacity was found to be 0.0596 mmol FeSO_4_/g for SMS (Figure 1D,E). The positive control drug NAC exhibited superior free radical scavenging capacity and reducing power compared to SMS.

### 3.3. Effects of HS on Rat H9c2 Cardiomyocytes

**Figure 2:**
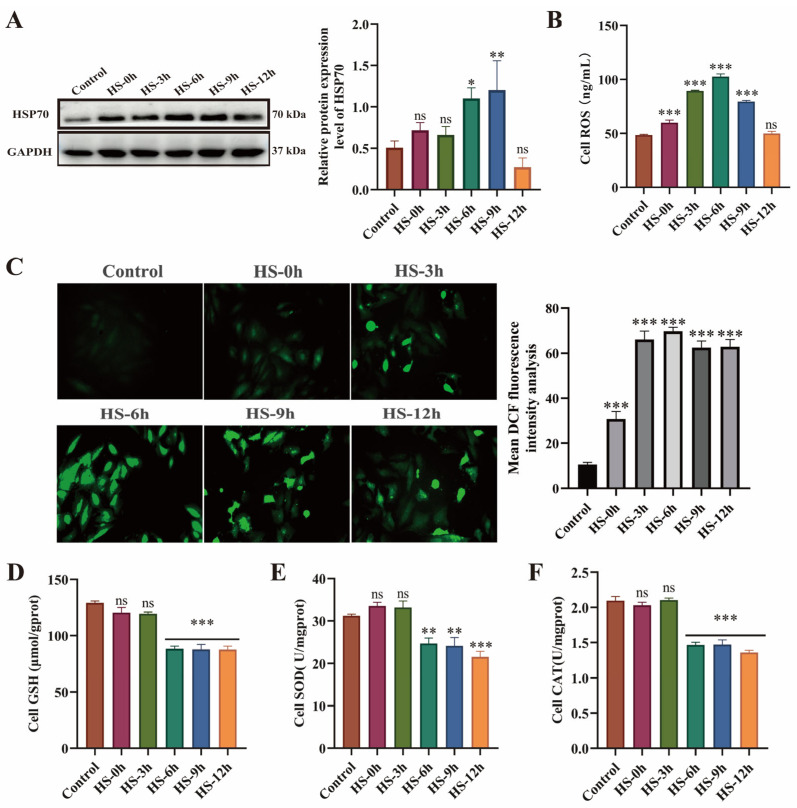
HS induces dysregulation of the heat shock response and oxidative stress injury in H9c2 cardiomyocytes. (**A**) HSP70 expression levels in H9c2 cells. (**B**,**C**) Intracellular ROS levels. (**D**–**F**) Activities of GSH, SOD, and CAT; * *p* < 0.05, ** *p* < 0.01, *** *p* < 0.001 vs. control group, n.s., not significant (*p* > 0.05) (n ≥ 3/group).

HSP70, a key biomarker of the HS response [37], exhibited a time-dependent upregulation during recovery, with significantly elevated expression levels observed at 6–9 h post-recovery (*p* < 0.05) (Figure 2A). Compared with the control group, intracellular ROS levels exhibited progressive accumulation during recovery, peaking at 6 h post-recovery (Figure 2B–C). Antioxidant enzyme activity assays revealed that GSH, SOD, and CAT activities were significantly reduced during 6–12 h recovery (*p* < 0.01), with values markedly lower than control levels (Figure 2D–F). These findings demonstrate that HS-induced myocardial cell injury primarily occurs during 6–12 h post-recovery, with the most severe damage observed at 6 h.

### 3.4. Protective Effects of SMS on Heat-Stressed H9c2 Cardiomyocytes

**Figure 3:**
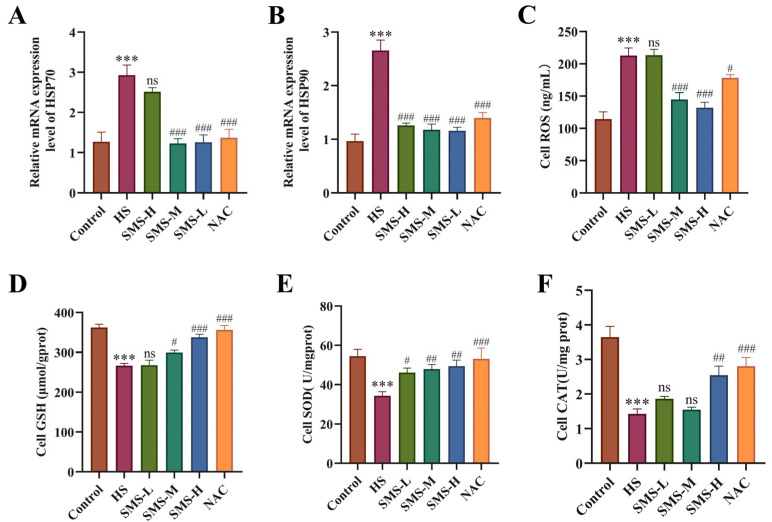
SMS attenuates HS-induced cellular injury in H9c2 cardiomyocytes by modulating heat shock proteins and enhancing antioxidant capacity. (**A**,**B**) mRNA expression levels of HSP70 and HSP90 in H9c2 cardiomyocytes. (**C**–**F**) Contents of ROS, GSH, SOD, and CAT in H9c2 cardiomyocytes. H9c2 cardiomyocytes were treated with SMS at concentrations of 500, 1000, and 2000 μg/mL or NAC (0.125 μM). *** *p* < 0.001 vs. control group; # *p* < 0.05, ## *p* < 0.01, ### *p* < 0.001 vs. HS group, n.s., not significant (*p* > 0.05) (n ≥ 3/group).

To investigate the protective effects of SMS against HS-induced injury in H9c2 cardiomyocytes, we examined changes in HSPs and oxidative stress markers. The results showed that compared with the control group, HS not only significantly promoted the increase of ROS (*p* < 0.01), but also markedly increased mRNA expression levels of both HSP70 and HSP90 (*p* < 0.01) (Figure 3A–C). Concurrently, HS markedly reduced the activities of GSH, SOD, and CAT (*p* < 0.01) (Figure 3D–F). Treatment with different doses of SMS or NAC not only significantly reduced intracellular ROS levels and heat shock protein expression but also restored antioxidant enzyme activities, with medium and high doses of SMS showing particularly significant improvements. These findings demonstrate that SMS effectively alleviates HS-induced oxidative imbalance in H9c2 cardiomyocytes, inhibits ROS overaccumulation, and regulates HSP homeostasis.

### 3.5. Effects of SMS on the Nrf2-HO-1 Pathway in Heat-Stressed Rat H9c2 Cardiomyocytes

**Figure 4:**
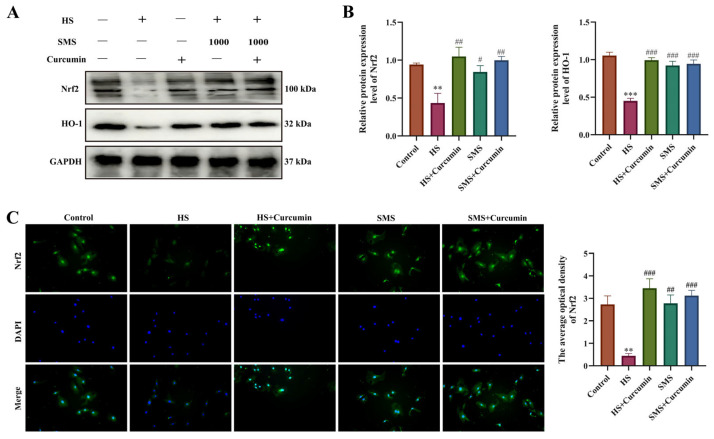
SMS attenuates HS-induced injury in H9c2 cardiomyocytes through activation of the Nrf2-HO-1 signaling pathway. (**A**) Representative Western blot images. (**B**) Quantitative analysis of Western blot results. (**C**) Immunofluorescence analysis of Nrf2 protein expression. H9c2 cardiomyocytes were treated with SMS (1000 μg/mL) or curcumin (2.5 μM). ** *p* < 0.01, *** *p* < 0.001 vs. control group; # *p* < 0.05, ## *p* < 0.01, ### *p* < 0.001 vs. HS group, n.s., not significant (*p* > 0.05) (n ≥ 3/group).

The Nrf2-HO-1 signaling pathway, a crucial antioxidant defense mechanism, was investigated to determine SMS’s protective effects against HS in H9c2 cardiomyocytes. Compared to controls, HS significantly downregulated Nrf2 and HO-1 protein expression (*p* < 0.01) while retaining Nrf2 primarily in the cytoplasm. Both SMS treatment and curcumin (Nrf2 agonist) intervention effectively reversed these effects (*p* < 0.01), promoting Nrf2 nuclear translocation and enhanced expression. Notably, no significant difference existed between SMS alone and SMS-curcumin combination therapy (*p* > 0.05), indicating that SMS specifically activates the Nrf2-HO-1 pathway for cardio-protection (Figure 4). These findings demonstrate that SMS mitigates HS damage through targeted Nrf2-HO-1 pathway modulation.

### 3.6. Effects of SMS on the Stub1-HSF1 Pathway in Heat-Stressed Rat H9c2 Cardiomyocytes

**Figure 5:**
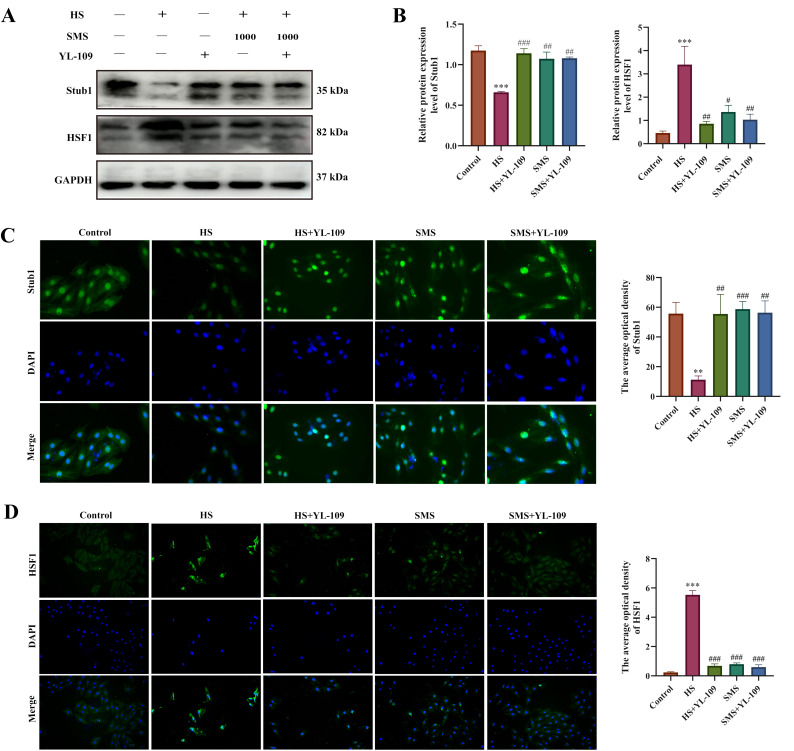
SMS attenuates HS-induced injury in H9c2 cardiomyocytes by suppressing the Stub1-HSF1 signaling pathway. (**A**) Representative Western blot images. (**B**) Quantitative analysis of Western blot results. (**C**) Immunofluorescence analysis of Stub1 expression. (**D**) Immunofluorescence analysis of HSF1 expression. H9c2 cardiomyocytes were treated with SMS (1000 μg/mL) or YL-109 (4 μM). ** *p* < 0.01, *** *p* < 0.001 vs. control group; # *p* < 0.05, ## *p* < 0.01, ### *p* < 0.001 vs. HS group, n.s., not significant (*p* > 0.05) (n ≥ 3/group).

The Stub1-HSF1 signaling pathway serves as a critical molecular switch that regulates the heat shock response and plays a central role in maintaining proteostasis during HS. To elucidate the effects of SMS on the Stub1-HSF1 regulatory network in heat-stressed H9c2 cardiomyocytes, we performed intervention experiments using YL-109, a specific Stub1 agonist. The results demonstrated that compared with the control group, HS significantly reduced Stub1 protein expression levels (*p* < 0.05), with synchronous decreases observed in both cytoplasmic and nuclear compartments, suggesting a systemic impairment of Stub1-mediated proteostatic regulation under thermal stress conditions. Concurrently, HSF1 expression was significantly elevated (*p* < 0.05) with marked nuclear translocation. Both YL-109 and SMS interventions effectively upregulated Stub1 expression (*p* < 0.05) while suppressing HSF1 overactivation (*p* < 0.05). Notably, no statistically significant difference was observed between SMS monotherapy and the SMS-YL-109 combination therapy (*p* > 0.05), suggesting that SMS likely exerts its cardioprotective effects through targeted modulation of the Stub1-HSF1 signaling pathway (Figure 5).

### 3.7. Effects of SMS on the Keap1/Nrf2/HO-1 Pathway in Heat-Stressed Rat Myocardium

**Figure 6:**
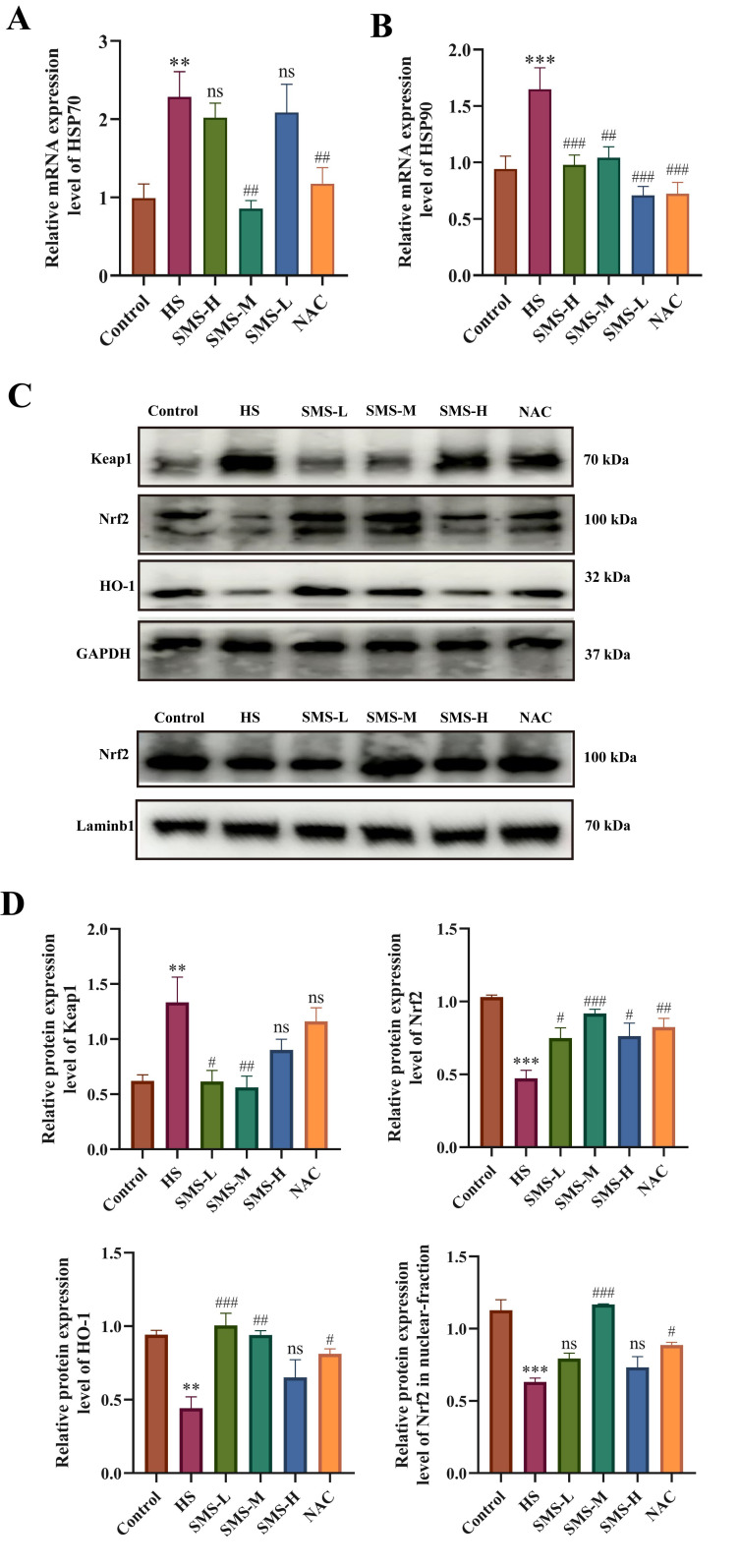
SMS attenuates HS-induced myocardial injury in rats through activation of the Keap1-Nrf2-HO-1 pathway and suppression of heat shock protein expression. (**A**,**B**) Effects of SMS on myocardial HSP70 and HSP90 mRNA expression levels under HS. (**C**) Representative Western blot images. (**D**) Quantitative analysis of Western blot results. Rats were treated with SMS at doses of 1.26, 2.52, and 5.04 g/kg or NAC (150 mg/kg). ** *p* < 0.01, *** *p* < 0.001 vs. control group; # *p* < 0.05, ## *p* < 0.01, ### *p* < 0.001 vs. HS group, n.s., not significant (*p* > 0.05) (n ≥ 5/group).

Our animal studies further demonstrated that SMS exerts multi-target cardioprotective effects against HS through modulation of the Keap1-Nrf2-HO-1 pathway. The results showed that SMS significantly attenuated HS-induced overexpression of HSP70 and HSP90 mRNA (*p* < 0.05) (Figure 6A,B). Notably, while HS markedly increased Keap1 protein expression (*p* < 0.05) and decreased both Nrf2 and HO-1 levels (*p* < 0.01) with reduced Nrf2 nuclear translocation compared to controls, SMS treatment effectively normalized these protein expression abnormalities (Figure 6C,D). These in vivo findings closely aligned with our cellular experiments, suggesting that SMS provides myocardial protection through coordinated regulation of both heat shock response and antioxidant defense systems.

### 3.8. Effects of SMS on the Stub1-HSF1 Pathway in Heat-Stressed Rat Myocardium

**Figure 7:**
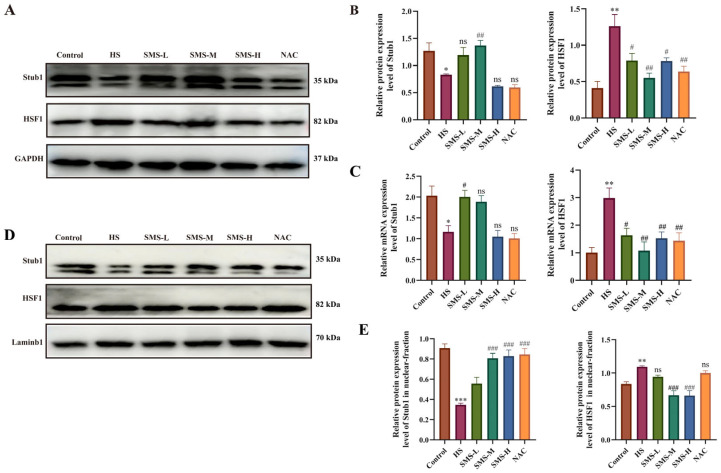
SMS attenuates HS-induced myocardial injury in rats by suppressing Stub1 and HSF1 expression and nuclear translocation. (**A**) Representative Western blot images of total Stub1 and HSF1 protein. (**B**) Quantitative analysis of total protein expression. (**C**) mRNA expression levels of Stub1 and HSF1. (**D**) Representative Western blot images of nuclear Stub1 and HSF1. (**E**) Quantitative analysis of nuclear protein content. Rats were treated with SMS at doses of 1.26, 2.52, and 5.04 g/kg or NAC (150 mg/kg). * *p* < 0.05, ** *p* < 0.01, *** *p* < 0.001 vs. control group; # *p* < 0.05, ## *p* < 0.01, ### *p* < 0.001 vs. HS group, n.s., not significant (*p* > 0.05) (n ≥ 3/group).

To elucidate the molecular mechanisms underlying SMS’s cardio-protection through modulation of the Stub1-HSF1 pathway, we systematically analyzed Stub1 and HSF1 expression at both the protein and gene levels in myocardial tissues using Western blot and qRT-PCR. The experimental results demonstrated remarkable consistency with our previous cellular findings: compared to the control group, HS significantly decreased Stub1 expression at both the mRNA and protein levels (including total and nuclear fractions) (*p* < 0.05) while conversely increasing HSF1 expression (both transcriptional and translational levels) (*p* < 0.05). Notably, SMS intervention effectively reversed these molecular alterations (*p* < 0.05), providing compelling evidence that SMS exerts its cardioprotective effects through targeted regulation of the Stub1-HSF1 signaling pathway (Figure 7).

## 4. Discussion

HS represents one of the most significant environmental challenges to human and animal health [38], particularly in tropical and subtropical regions worldwide [39]. Substantial evidence indicates that HS-induced physiological damage typically manifests not during the initial exposure phase, but rather during the subsequent recovery or delayed phases, a phenomenon consistent with numerous research and clinical observations [33,40]. Our study demonstrates that HS significantly increases intracellular ROS levels in cardiomyocytes. When ROS overproduction exceeds the buffering capacity of endogenous antioxidant defense systems, pathological accumulation occurs, ultimately leading to oxidative stress. HS significantly decreased the activities of key antioxidant enzymes including GSH, SOD, and CAT in cardiomyocytes. These findings are consistent with previous reports: Chen et al. observed significantly increased ROS levels in cardiac tissue at 6 h post-HS, accompanied by progressively reduced SOD and CAT activities up to 12 h [41]. Similarly, Wang et al. demonstrated in an in vitro HS model that both SOD and GSH were markedly decreased while ROS was significantly elevated at 6 h after HS cessation [42]. This collective evidence confirms the characteristic delayed onset (typically peaking at 6–12 h post-stress) of oxidative cardiac injury induced by HS. The resulting oxidative stress triggers the production and release of HSPs, which serve as a protective mechanism against ROS-mediated cellular damage [43]. Importantly, the accumulation of HSP70 has been established as a reliable biomarker for detecting HS-induced injury [37]. Kaushik et al. demonstrated that HS stimulation significantly increased HSP70 mRNA expression in cultured peripheral blood mononuclear cells (PBMCs) in a temperature- and time-dependent manner compared to control conditions [44]. Similarly, Pawar et al. reported time-dependent elevation of HSP70 mRNA levels in PBMCs during 0–4 h of HS exposure, followed by gradual decline [45]. Our current study revealed parallel findings in cardiomyocytes, showing progressive accumulation of HSP70 during post-stress recovery, with particularly significant upregulation at 6–9 h. These consistent observations across different experimental systems collectively indicate that compromised antioxidant defenses and disrupted HSP homeostasis play pivotal roles in HS-induced myocardial injury.

Current clinical strategies for managing HS (including physical cooling, fluid replacement, antipyretics, and organ support therapy) can alleviate symptoms but show limited efficacy in regulating HS-induced oxidative stress. Therefore, developing effective and safe anti-HS medications is of significant importance. TCM, with its multi-target approach and low toxicity characteristics, demonstrates unique potential in HS prevention and control. SMS, a classic Qi-invigorating and Yin-nourishing formula, is widely used in TCM and in some Asian countries for the adjunctive treatment of cardiovascular conditions such as heart failure, angina pectoris, and myocardial infarction [24]. It should be noted that SMS is not yet specifically recommended in major international cardiovascular guidelines (e.g., ACC/AHA or ESC guidelines); however, its clinical application is supported by a body of evidence from TCM clinical practice and pharmacological studies [24,46,47]. Nevertheless, its protective effects against HS-induced myocardial injury and the underlying mechanisms remain poorly understood. Our study identified that SMS contains abundant polysaccharides, flavonoids, and saponins, with total polysaccharides being the most predominant component followed by total saponins. Previous studies have confirmed that these bioactive compounds exhibit significant anti-inflammatory, antioxidant, and immunomodulatory effects [48,49,50,51]. Specifically, Wang et al. demonstrated that ginsenoside Rg1 could alleviate hydrogen peroxide-induced neuronal oxidative damage by reducing ROS and MDA levels [52]. Furthermore, Shin et al. revealed that ginsenoside Re concentration-dependently inhibited UV-B irradiation-induced ROS accumulation in HaCaT cells while modulating the GSH-SOD system activity [53]. Furthermore, our in vitro antioxidant assays demonstrated that SMS effectively scavenges DPPH, ABTS, and OH^−^ radicals while exhibiting Fe^3+^ reducing capacity, indicating direct antioxidant activity. These findings are supported by studies from Chen et al. [54] and Wang et al. [55], although variations in antioxidant potency were observed among different polar fractions and formulations (Yiqi Fumai freeze-dried powder), potentially attributable to compositional differences or preparation techniques. These results provide a theoretical foundation for further pharmacological investigation and clinical application of SMS. Our study revealed that SMS intervention significantly downregulated HSP70 and HSP90 expression in both H9c2 cardiomyocytes and heat-stressed rat hearts, while markedly elevating myocardial levels of GSH, SOD, and CAT. This cardioprotective effect may be mediated through the regulatory actions of saponin constituents (ginsenosides) present in the formula. Supporting evidence comes from Feng’s research demonstrating that total ginsenosides improved the physiological status of heat-stressed cattle by enhancing the antioxidant defense system (reducing MDA while increasing GSH and SOD levels) [56]. Further corroboration comes from Liu et al.’s findings that ginsenoside Rg3 attenuated testicular oxidative damage and inhibited seminiferous tubule atrophy and apoptosis [57]. Collectively, these results suggest that SMS’s cardioprotection against HS likely involves modulation of oxidative stress pathways by its active components, particularly saponins and polysaccharides.

The present study demonstrated through both cellular and animal experiments that HS significantly upregulates Keap1 expression while downregulating Nrf2 and its downstream target gene HO-1 in myocardial tissues. These findings are consistent with previous reports: Liu et al. [58] observed significant reductions in Nrf2 and HO-1 expression in skeletal muscle cells under HS, while Wang [59] demonstrated impaired nuclear translocation of Nrf2 in neutrophils following heat exposure. Notably, our experimental results revealed that both the Nrf2 agonist curcumin and the TCM compound SMS effectively reversed HS-induced suppression of the Nrf2 signaling pathway and significantly promoted Nrf2 nuclear translocation. Based on our preliminary findings, we hypothesize that the antioxidant effects of SMS may involve scavenging excessive ROS, thereby indirectly modulating the activation of the Keap1-Nrf2-HO-1 signaling pathway. Specifically, SMS likely regulates this pathway through ROS elimination, as ROS can oxidatively modify critical cysteine residues in Keap1, inducing conformational changes that weaken its binding affinity for Nrf2. This molecular mechanism facilitates Nrf2 nuclear translocation and subsequent activation of downstream antioxidant genes [60]. The current study not only elucidates the pivotal regulatory role of the Keap1-Nrf2-HO-1 signaling pathway in HS-induced myocardial injury, but also provides novel theoretical evidence for understanding the cardioprotective mechanisms of SMS.

This study systematically elucidates the pivotal regulatory role and molecular mechanisms of the Stub1-HSF1 signaling pathway in HS-induced myocardial injury. By establishing both in vitro and in vivo HS models, we identified that HS specifically downregulates Stub1 expression in cardiomyocytes-a phenomenon distinct from classical ischemia/hypoxia injury models [61], suggesting that different stress stimuli may disrupt myocardial homeostasis through unique molecular pathways. Our study further demonstrated that under HS conditions, decreased Stub1 expression leads to significantly increased HSF1 nuclear translocation, resulting in excessive expression of downstream HSPs such as HSP70. This regulatory mechanism involves Stub1’s dual functionality: as an E3 ubiquitin ligase that promotes HSF1 degradation [62,63], and through its molecular chaperone activity that modulates HSF1 conformational changes [64,65]. Importantly, SMS effectively regulated the Stub1-HSF1 pathway, inhibiting abnormal HSF1 nuclear translocation and reducing HSP70 overexpression to an extent comparable with the Stub1 agonist YL-109. These findings not only confirm the critical role of the Stub1-HSF1 pathway in HS-induced myocardial injury, but also reveal that SMS ameliorates HS-mediated cardiac damage through targeted regulation of this pathway, providing important theoretical foundations for related drug development.

This study provides the first demonstration that SMS exerts cardioprotective effects against HS -induced myocardial injury through dual mechanisms: activation of the Nrf2-HO-1 antioxidant pathway and suppression of the Stub1-HSF1 heat shock pathway. This discovery not only provides a novel pharmacological rationale for the clinical application of SMS but also reveals the great potential of synergistic therapeutic strategies targeting multiple stress pathways (oxidative and proteotoxic stress). A key strength of this study lies in the rigorous mutual validation through both in vivo and in vitro models, which systematically elucidates this previously unrecognized mechanism. However, several limitations should be acknowledged. First, although we confirmed that Stub1 is a critical regulator of HSF1 activity and the downstream heat shock response, and previous studies have indicated its involvement in oxidative stress regulation [66,67], we have not yet directly demonstrated through gene knockout technology that Stub1 serves as the central bridging molecule connecting these two pathways in our model. Second, the potential crosstalk between the Nrf2 and HSF1 pathways, as well as their hierarchical relationship, remains to be fully elucidated. Furthermore, while the current research primarily focuses on preclinical mechanistic exploration, its translational applicability requires further validation through subsequent preclinical pharmacodynamic studies and biomarker development.

## 5. Conclusions

This study reveals the multi-target protective mechanisms of SMS against HS-induced myocardial injury. As the main active components of this formula, polysaccharides and saponins demonstrate remarkable antioxidant capacity. Our findings show that HS-induced myocardial injury reaches peak severity between 6 and 12 h post-exposure, with maximal damage occurring at 6 h. SMS treatment significantly mitigates this injury through coordinated actions: reducing oxidative stress by decreasing ROS levels while enhancing GSH, SOD and CAT activities; downregulating the excessive expression of HSP70 and HSP90; and modulating both the Keap1-Nrf2-HO-1 and Stub1-HSF1 signaling pathways. These results provide molecular-level evidence supporting the cardioprotective effects of SMS.

## Figures and Tables

**Table 1:** Chemical composition analysis of SMS.

SMS	Total Polysaccharides	Total Saponins	Total Flavonoids
Precision (RSD)	0.61%	1.54%	3.56%
Stability (RSD)	0.19%	3.34%	3.14%
Repeatability (RSD)	3.31%	2.40%	4.20%
Spike Recovery (Mean Recovery)	96.69%	106.06%	93.73%
Spike Recovery (RSD)	3.69%	2.61%	1.63%
Validation Test (RSD)	3.37%	3.35%	1.40%
Content	57.41%	10.98%	0.42%

## Data Availability

The datasets used and/or analyzed during the current study are available from the corresponding author upon reasonable request.

## Abbreviations

Sheng Mai San, SMS; heat stress, HS; traditional Chinese medicine, TCM; reactive oxygen species, ROS; catalase, CAT; superoxide dismutase, SOD; glutathione, GSH; Nuclear factor erythroid 2-related factor 2, Nrf2; Kelch-like ECH-associated protein 1, Keap1; Heme oxygenase-1, HO-1; N-acetylcysteine, NAC; heat shock factor 1, HSF1; heat shock protein, HSP; quantitative real-time polymerase chain reaction, qRT-PCR.

## References

[1] Cramer M.N., Gagnon D., Laitano O., Crandall C.G. (2022). Human temperature regulation under heat stress in health, disease, and injury. Physiol. Rev..

[2] Becker C.A., Collier R.J., Stone A.E. (2020). Invited review: Physiological and behavioral effects of heat stress in dairy cows. J. Dairy Sci..

[3] Khan M.Z., Khan A., Chen W., Chai W., Wang C. (2024). Advancements in Genetic Biomarkers and Exogenous Antioxidant Supplementation for Safeguarding Mammalian Cells against Heat-Induced Oxidative Stress and Apoptosis. Antioxidants.

[4] Curriero F.C., Heiner K.S., Samet J.M., Zeger S.L., Strug L., Patz J.A. (2002). Temperature and mortality in 11 cities of the eastern United States. Am. J. Epidemiol..

[5] Gu Z.T. (2014). Mitochondrial Pathway-Mediated Apoptosis Induced by Heat Stress in Human Umbilical Vein Endothelial Cells. Ph.D. Thesis.

[6] Huang J., Li G.X., Xu G.Z. Impact of temperature variation on years of life lost in a typical subtropical city in Southern China: A time-series analysis. Proceedings of the 2017 Conference on Environment and Public Health & Annual Meetings of the Environmental Medicine and Health Branch of the Chinese Society for Environmental Sciences and the Biochemical and Molecular Toxicology Professional Committee of the Chinese Society of Toxicology.

[7] Yin J.F. (2011). Impacts of Summer Outdoor Thermal Environment on Human Health and Associated Assessment Techniques. Master’s Thesis.

[8] Luo X.R., Li S.H., Xu G.Z. Wuhan Center for Disease Control and Prevention. Effect of summer heat waves on mortality among residents in Wuhan. Proceedings of the Chinese Meteorological Society.

[9] Wu Y.N. (2022). Study on the Inhibitory Effects of Quercetin Marigold Flavonoids on Heat Stress-Induced Cardiac Injury in Broilers. Master’s Thesis.

[10] Murakami S., Kusano Y., Okazaki K., Akaike T., Motohashi H. (2023). NRF2 signalling in cytoprotection and metabolism. Br. J. Pharmacol..

[11] Zhang J., Zhang M., Tatar M., Gong R. (2025). Keap1-independent Nrf2 regulation: A novel therapeutic target for treating kidney disease. Redox Biol..

[12] Huenchuguala S., Segura-Aguilar J. (2024). Natural Compounds That Activate the KEAP1/Nrf2 Signaling Pathway as Potential New Drugs in the Treatment of Idiopathic Parkinson’s Disease. Antioxidants.

[13] Adinolfi S., Patinen T., Jawahar Deen A., Pitkänen S., Härkönen J., Kansanen E., Küblbeck J., Levonen A.L. (2023). The KEAP1-NRF2 pathway: Targets for therapy and role in cancer. Redox Biol..

[14] Ishitsuka Y., Ogawa T., Roop D. (2020). The KEAP1/NRF2 Signaling Pathway in Keratinization. Antioxidants.

[15] Lv T., Zhang K.R. (2011). Research Advances in the Co-chaperone Protein STUB1 and Its Functions. Chin. Pharmacol. Bull..

[16] Rosser M.F., Washburn E., Muchowski P.J., Patterson C., Cyr D.M. (2007). Chaperone functions of the E3 ubiquitin ligase CHIP. J. Biol. Chem..

[17] Sioen I., Hacquebard M., Hick G., Maindiaux V., Larondelle Y., Carpentier Y.A., De Henauw S. (2009). Effect of ALA-enriched food supply on cardiovascular risk factors in males. Lipids.

[18] Samali A., Holmberg C.I., Sistonen L., Orrenius S. (1999). Thermotolerance and cell death are distinct cellular responses to stress: Dependence on heat shock proteins. FEBS Lett..

[19] Morimoto R.I. (1998). Regulation of the heat shock transcriptional response: Cross talk between a family of heat shock factors, molecular chaperones, and negative regulators. Genes. Dev..

[20] Dai Q., Zhang C., Wu Y., McDonough H., Whaley R.A., Godfrey V., Li H.H., Madamanchi N., Xu W., Neckers L. (2003). CHIP activates HSF1 and confers protection against apoptosis and cellular stress. EMBO J..

[21] Ma W.F. (2008). Effects of Sheng Mai San on Endotoxin Levels and Cytokines in Liver Failure. Master’s Thesis.

[22] Jiang Y., He Q., Zhang T., Xiang W., Long Z., Wu S. (2020). Exploring the mechanism of Shengmai Yin for coronary heart disease based on systematic pharmacology and chemoinformatics. Biosci. Rep..

[23] Guo J.N., Chun L.X., Fan L.H. (2025). Research Progress on Sheng Mai San in the Treatment of Cardiovascular Diseases. J. Liaoning Univ. Tradit. Chin. Med..

[24] Yang Y., Tian Y., Hu S., Bi S., Li S., Hu Y., Kou J., Qi J., Yu B. (2017). Extract of Sheng-Mai-San Ameliorates Myocardial Ischemia-Induced Heart Failure by Modulating Ca^2+^-Calcineurin-Mediated Drp1 Signaling Pathways. Int. J. Mol. Sci..

[25] Zhang X., Jia Y., Yuan Z., Wen Y., Zhang Y., Ren J., Ji P., Yao W., Hua Y., Wei Y. (2022). Sheng Mai San ameliorated heat stress-induced liver injury via regulating energy metabolism and AMPK/Drp1-dependent autophagy process. Phytomedicine.

[26] Zhong G., Chen J., Li Y., Han Y., Wang M., Nie Q., Xu M., Zhu Q., Chang  X., Wang L. (2024). Ginsenoside Rg3 attenuates myocardial ischemia/reperfusion-induced ferroptosis via the keap1/Nrf2/GPX4 signaling pathwayGinsenoside Rg3 ameliorates acute pancreatitis by activating the NRF2/HO-1-mediated ferroptosis pathway. BMC Complement. Med. Ther..

[27] Ni S., Qian Z., Yuan Y., Li D., Zhong Z., Ghorbani F., Zhang X., Zhang F., Zhang Z., Liu Z. (2020). Schisandrin A restrains osteoclastogenesis by inhibiting reactive oxygen species and activating Nrf2 signalling. Cell Prolif..

[28] Chen S.L., Liu An Li Q., Shan T.X., Zhu G.W., Sun Y., Dai Y.T., Zhang J., Zhang T.J., Liu C.X. (2016). Research Strategies for Standard Decoctions of Chinese Herbal Pieces. China J. Chin. Mater. Med..

[29] Deng Z., Jing W.G., Wang S.H., Jiao M.J., Zhang Q., Zhou H.Y., Zhang J., Liu A. (2019). Research Progress and Discussion on Standard Decoctions of Chinese Herbal Pieces. China J. Chin. Mater. Med..

[30] Zhang C.H., Yun Y.H., Fan W., Liang Y.Z., Yu Y., Tang W.X. (2015). Rapid analysis of polysaccharides contents in Glycyrrhiza by near infrared spectroscopy and chemometrics. Int. J. Biol. Macromol..

[31] Basu P., Hornung R.S., Averitt D.L., Maier C. (2019). Euphorbia bicolor (*Euphorbiaceae*) Latex Extract Reduces Inflammatory Cytokines and Oxidative Stress in a Rat Model of Orofacial Pain. Oxid. Med. Cell Longev..

[32] Qu Z.Y., Zhao J.H., Liu H.Q., Yao C.L., Wang Y.P. (2012). Determination of Total Ginsenosides in Preserved Ginseng by Colorimetric Method. J. Ginseng Res..

[33] Geng Y., Ma Q., Liu Y.N., Peng N., Yuan F.F., Li X.G., Li M., Wu Y.S., Li B.L., Song W.B. (2015). Heatstroke induces liver injury via IL-1β and HMGB1-induced pyroptosis. J. Hepatol..

[34] Zhao Q.L., Fujiwara Y., Kondo T. (2006). Mechanism of cell death induction by nitroxide and hyperthermia. Free Radic. Biol. Med..

[35] Zhang X.S. (2022). Protective Effects and Mechanisms of Sheng Mai San Against Heat Stress Induced Liver Injury in Rats. Ph.D. Thesis.

[36] Yang L., Duan Z., Liu X., Yuan Y. (2018). N-acetyl-l-cysteine ameliorates the PM2.5-induced oxidative stress by regulating SIRT-1 in rats. Environ. Toxicol. Pharmacol..

[37] Tedeschi J.N., Kennington W.J., Berry O., Whiting S., Meekan M., Mitchell N.J. (2015). Increased expression of Hsp70 and Hsp90 mRNA as biomarkers of thermal stress in loggerhead turtle embryos (Caretta Caretta). J. Therm. Biol..

[38] Delandmeter M., de Faccio Carvalho P.C., Bremm C., Dos Santos Cargnelutti C., Bindelle J., Dumont B. (2024). Integrated crop and livestock systems increase both climate change adaptation and mitigation capacities. Sci. Total Environ..

[39] Patra A.K., Kar I. (2021). Heat stress on microbiota composition, barrier integrity, and nutrient transport in gut, production performance, and its amelioration in farm animals. J. Anim. Sci. Technol..

[40] Davis B.C., Tillman H., Chung R.T., Stravitz R.T., Reddy R., Fontana R.J., McGuire B., Davern T., Lee W.M. (2017). Acute Liver Failure Study Group. Heat stroke leading to acute liver injury & failure: A case series from the Acute Liver Failure Study Group. Liver Int..

[41] Chen C.M., Hou C.C., Cheng K.C., Tian R.L., Chang C.P., Lin M.T. (2006). Activated protein C therapy in a rat heat stroke model. Crit. Care Med..

[42] Wang M., Liu Y., Li H., Wang X.X., Liu H. (2024). Protective Effects and Mechanisms of Paeoniflorin Against Heat Stress-Induced Myocardial Injury. Acta Nutr. Sin..

[43] Dröge W. (2002). Free radicals in the physiological control of cell function. Physiol. Rev..

[44] Kaushik R., Goel A., Rout P.K. (2022). Differential expression and regulation of HSP70 gene during growth phase in ruminants in response to heat stress. Sci. Rep..

[45] Pawar S.S., Kurade N.P., Bhendarkar M.P., Bhosale S.V., Nirmale A.V., Kochewad S.A. (2023). Modulation of heat shock protein 70 (HSP70) gene expression ex vivo in response to heat stress in chicken. Anim. Biotechnol..

[46] Hou Y., He Z., Han Y., Zhang T., Wang S., Wang X., Mao J. (2023). Mechanism of new optimized Sheng-Mai-San Formula to regulate cardiomyocyte apoptosis through NMDAR pathway. Heliyon.

[47] Mo W.L., Chai C.Z., Kou J.P., Yan Y.Q., Yu B.Y. (2015). Sheng-Mai-San attenuates contractile dysfunction and structural damage induced by chronic intermittent hypoxia in mice. Chin. J. Nat. Med..

[48] Yu Y., Shen M., Song Q., Xie J. (2018). Biological activities and pharmaceutical applications of polysaccharide from natural resources: A review. Carbohydr. Polym..

[49] Singh S., Gupta P., Meena A., Luqman S. (2020). Acacetin, a flavone with diverse therapeutic potential in cancer, inflammation, infections and other metabolic disorders. Food Chem. Toxicol..

[50] Hostetler G.L., Ralston R.A., Schwartz S.J. (2017). Flavones: Food Sources, Bioavailability, Metabolism, and Bioactivity. Adv. Nutr..

[51] Góral I., Wojciechowski K. (2020). Surface activity and foaming properties of saponin-rich plants extracts. Adv. Colloid. Interface Sci..

[52] Wang Y., Liu Q., Xu Y., Zhang Y., Lv Y., Tan Y., Jiang N., Cao G., Ma X., Wang J. (2016). Ginsenoside Rg1 Protects against Oxidative Stress-induced Neuronal Apoptosis through Myosin IIA-actin Related Cytoskeletal Reorganization. Int. J. Biol. Sci..

[53] Shin D., Moon H.W., Oh Y., Kim K., Kim D.D., Lim C.J. (2018). Defensive Properties of Ginsenoside Re against UV-B-Induced Oxidative Stress through Up-Regulating Glutathione and Superoxide Dismutase in HaCaT Keratinocytes. Iran. J. Pharm. Res..

[54] Chen H.F., Ji J., Yu B.Y. (2016). Antioxidant Effects of Extracts from Panax ginseng, Ophiopogon japonicus, and Schisandra chinensis in Shengmai San In Vitro. Chin. J. Exp. Tradit. Med. Formulae.

[55] Ichikawa H., Wang X., Konishi T. (2003). Role of component herbs in antioxidant activity of shengmai san--a traditional Chinese medicine formula preventing cerebral oxidative damage in rat. Am. J. Chin. Med..

[56] Feng J.J. (2022). Effects of Panax ginseng Total Saponins on Weight Gain, Hematological Parameters, and Rumen Microbiota in Beef Cattle Under Heat Stress. Master’s Thesis.

[57] Liu W. (2019). Preparation and Isolation of Ginseng Secondary Saponins and the Protective Effect of Ginsenoside Rg3 on Scrotal Heat Stress Injury in Mice. Master’s Thesis.

[58] Liu Y., Jia Y.Q., Li C.C., Mao H.D., Liu S.Y., Shan Y. (2025). Dexmedetomidine Attenuates Heat Stress-Induced Oncosis in Human Skeletal Muscle Cells via Activation of the Nrf2/HO-1 Pathway. J. S. Med. Univ..

[59] Wang C. (2024). L-Arginine Attenuates Heat Stress-Induced Neutrophil Apoptosis in Dairy Cows via the Nrf2/ROS Pathway. Master’s Thesis.

[60] Kobayashi A., Kang M.I., Okawa H., Ohtsuji M., Zenke Y., Chiba T., Igarashi K., Yamamoto M. (2004). Oxidative stress sensor Keap1 functions as an adaptor for Cul3-based E3 ligase to regulate proteasomal degradation of Nrf2. Mol. Cell Biol..

[61] Zhao B., Sun G., Feng G., Duan W., Zhu X., Chen S., Hou L., Jin Z., Yi D. (2012). Carboxy terminus of heat shock protein (HSP) 70-interacting protein (CHIP) inhibits HSP70 in the heart. J. Physiol. Biochem..

[62] Sha Y., Rao L., Settembre C., Ballabio A., Eissa N.T. (2017). STUB1 regulates TFEB-induced autophagy-lysosome pathway. EMBO J..

[63] Nadel C.M., Thwin A.C., Callahan M., Lee K., Connelly E., Craik C.S., Southworth D.R., Gestwicki J.E. (2023). The E3 Ubiquitin Ligase, CHIP/STUB1, Inhibits Aggregation of Phosphorylated Proteoforms of Microtubule-associated Protein Tau (MAPT). J. Mol. Biol..

[64] Huang C.Y., Kuo W.W., Lo J.F., Ho T.J., Pai P.Y., Chiang S.F., Chen P.Y., Tsai F.J., Tsai C.H., Huang C.Y. (2016). Doxorubicin attenuates CHIP-guarded HSF1 nuclear translocation and protein stability to trigger IGF-IIR-dependent cardiomyocyte death. Cell Death Dis..

[65] Qian S.B., McDonough H., Boellmann F., Cyr D.M., Patterson C. (2006). CHIP-mediated stress recovery by sequential ubiquitination of substrates and Hsp70. Nature.

[66] Tsvetkov P., Adamovich Y., Elliott E., Shaul Y. (2011). E3 ligase STUB1/CHIP regulates NAD(P)H:quinone oxidoreductase 1 (NQO1) accumulation in aged brain, a process impaired in certain Alzheimer disease patients. J. Biol. Chem..

[67] Kim S.A., Yoon J.H., Kim D.K., Kim S.G., Ahn S.G. (2005). CHIP interacts with heat shock factor 1 during heat stress. FEBS Lett..

